# Serum carotenoids and vitamins and risk of cervical dysplasia from a case–control study in Japan

**DOI:** 10.1038/sj.bjc.6690834

**Published:** 1999-12

**Authors:** C Nagata, H Shimizu, H Yoshikawa, K Noda, S Nozawa, A Yajima, S Sekiya, H Sugimori, Y Hirai, K Kanazawa, M Sugase, T Kawana

**Affiliations:** Department of Public Health, Gifu University School of Medicine, 40 Tsukasa-machi, Gifu 500-8705, Japan; Department of Obstetrics and Gynecology, University of Tokyo, Tokyo, Japan; Department of Obstetrics and Gynecology, Kinki University, Osaka, Japan; Department of Obstetrics and Gynecology, Keio University, Tokyo, Japan; Department of Obstetrics and Gynecology, Tohoku University, Miyagi, Japan; Department of Obstetrics and Gynecology, University of Chiba, Chiba, Japan; Department of Obstetrics and Gynecology, Saga Medical School, Saga, Japan; Department of Obstetrics and Gynecology, Cancer Institute Hospital, Tokyo, Japan; Department of Obstetrics and Gynecology, University of the Ryukyus, Okinawa, Japan; Department of Obstetrics and Gynecology, Nagano Red Cross Hospital, Nagano, Japan; Department of Obstetrics and Gynecology, Tokyo University Branch Hospital, Tokyo, Japan

**Keywords:** cervical dysplasia, carotenoids, human papillomavirus, diet

## Abstract

The relationships between risk of cervical dysplasia and dietary and serum carotenoids and vitamins were investigated in a case–control study. Cases were 156 women who attended Papanicolaou test screening in nine institutes affiliated with Japan Study Group of Human Papillomavirus (HPV) and Cervical Cancer and had cervical dysplasia newly histologically confirmed. Age-matched controls were selected from women with normal cervical cytology attending the same clinic. Blood sample and cervical exfoliated cells were obtained for measuring serum retinol, α-carotene, β-carotene, zeaxanthin/lutein, cryptoxanthin, lycopene and α-tocopherol and for HPV detection. Higher serum level of α-carotene was significantly associated with decreased risk of cervical dysplasia after controlling for HPV infection and smoking status (odds ratio (OR) = 0.16, 95% confidence interval (CI) 0.04–0.62 for the highest as compared with the lowest tertile). Decreased risk for the highest tertile of serum lycopene (OR = 0.28) was marginally significant. Decreased risks observed for the highest tertiles of β-carotene (OR = 0.65) and zeaxanthin/lutein (OR = 0.53), were not statistically significant. © 1999 Cancer Research Campaign


*British Journal of Cancer* (1999) **81**(7), 1234–1237
© 1999 Cancer Research Campaign
Article no. bjoc.1999.0834

### Serum carotenoids and vitamins and risk of cervical dysplasia from a case–control study in Japan

**C Nagata[1], H Shimizu[1], H Yoshikawa[2], K Noda[3], S Nozawa[4], A Yajima[5], S Sekiya[6], H Sugimori[7], Y Hirai[8], K Kanazawa[9], M Sugase[10] and T Kawana[11]**

[1]Department of Public Health, Gifu University School of Medicine, 40 Tsukasa-machi, Gifu 500-8705, Japan; [2]Department of Obstetrics and Gynecology, University of Tokyo, Tokyo, Japan; [3]Department of Obstetrics and Gynecology, Kinki University, Osaka, Japan; [4]Department of Obstetrics and Gynecology, Keio University, Tokyo, Japan; [5]Department of Obstetrics and Gynecology, Tohoku University, Miyagi, Japan; [6]Department of Obstetrics and Gynecology, University of Chiba, Chiba, Japan; [7]Department of Obstetrics and Gynecology, Saga Medical School, Saga, Japan; [8]Department of Obstetrics and Gynecology, Cancer Institute Hospital, Tokyo, Japan; [9]Department of Obstetrics and Gynecology, University of the Ryukyus, Okinawa, Japan; [10]Department of Obstetrics and Gynecology, Nagano Red Cross Hospital, Nagano, Japan; [11]Department of Obstetrics and Gynecology, Tokyo University Branch Hospital, Tokyo, Japan

**Summary** The relationships between risk of cervical dysplasia and dietary and serum carotenoids and vitamins were investigated in a case–control study. Cases were 156 women who attended Papanicolaou test screening in nine institutes affiliated with Japan Study Group of Human Papillomavirus (HPV) and Cervical Cancer and had cervical dysplasia newly histologically confirmed. Age-matched controls were selected from women with normal cervical cytology attending the same clinic. Blood sample and cervical exfoliated cells were obtained for measuring serum retinol, α-carotene, β-carotene, zeaxanthin/lutein, cryptoxanthin, lycopene and α-tocopherol and for HPV detection. Higher serum level of α-carotene was significantly associated with decreased risk of cervical dysplasia after controlling for HPV infection and smoking status (odds ratio (OR) = 0.16, 95% confidence interval (CI) 0.04–0.62 for the highest as compared with the lowest tertile). Decreased risk for the highest tertile of serum lycopene (OR = 0.28) was marginally significant. Decreased risks observed for the highest tertiles of β-carotene (OR = 0.65) and zeaxanthin/lutein (OR = 0.53), were not statistically significant. © 1999 Cancer Research Campaign

**Keywords:** cervical dysplasia; carotenoids; human papillomavirus; diet

It has been suggested that nutritional factors may affect the risk of cervical cancer. Numerous studies examined the relationships between vitamins, carotenoids and cervical neoplasia (Schneider and Shah, 1989; Potishman and Brinton, 1996). We previously reported decreased serum retinol levels in women with cervical dysplasia as compared with age-matched controls (Shimizu et al, 1996). However, most other studies have found no association of risk of cervical neoplasia with either dietary or serum retinol (Marshall et al, 1983; Harris et al, 1986; Brock et al, 1988; La Vecchia et al, 1988; Palan et al, 1988, 1991; Verreault et al, 1989; Cuzick et al, 1990; Ziegler et al, 1990; De Vet et al, 1991; Herrero et al, 1991). Relationships of dietary carotenoids to risk of cervical neoplasia have been inconsistent in previous studies (Marshall et al, 1983; Harris et al, 1986; Brock et al, 1988; La Vecchia et al, 1988; Palan et al, 1988; Cuzick et al, 1990; Ziegler et al, 1990; De Vet et al, 1991; Herrero et al, 1991; VanEenwyk et al, 1991). However, the results from serologic data have more consistently suggested a reduced risk associated with high serum carotenoid level (Harris et al, 1986; Palan et al, 1988; Brock et al, 1988; Verreault et al, 1989; Potishman et al, 1991; VanEenwyk et al, 1991; Batieha et al, 1993).

*Received 12 November 1998*
*Revised 8 April 1999*
*Accepted 13 April 1999*

*Correspondence to:* C Nagata

Epidemiological and laboratory evidence has supported the conclusion that human papillomavirus (HPV) infection is a primary causative agent for cervical cancer (Herrero, 1996). Nutritional factors may be only correlates of human papillomavirus (HPV) infection. Early epidemiological studies of HPV infection were limited by poorly validated detection method, but recently improved polymerase chain reaction (PCR)-based detection affords a greater sensitivity and specificity for a broad spectrum of HPV types (Schiffman, 1992). We determined HPV infection by PCR-based assays and evaluated the relationships between dietary and serum micronutrients and cervical dysplasia taking account of potential confounding effects of HPV infection and other factors.

### MATERIALS AND METHODS

Cases and controls for the present study were derived from women attending Papanicolaou test screening at nine institutes affiliated to the Japan Study Group of HPV and Cervical Cancer between June 1995 and July 1996. They returned to the institute about 1 week later to receive the result of the test. For women with abnormal findings, colposcopic examination was performed at that time. A total of 167 women aged 55 years or younger with newly histologically confirmed cervical dysplasia formed the case group. All histological diagnoses were reviewed by one pathologist. Their diagnoses were 94 mild, 40 moderate and 33 severe dysplasia. For each case, an institute- and age- (within 5 years) matched control was selected from women with normal cervical cytology. The first eligible match (if she refused to participate, the next eligible match) after the identification of case was approached. About 90% of the first eligible controls agreed to participate in the study.

A self-administered questionnaire including demographic factors, smoking and drinking habits, diet, reproductive history and sexual behaviour was distributed to each case and control when she visited the institute to know the result of Papanicolaou test. Diet was assessed by asking about the intake amounts of 22 foods that are retinol- and carotene-rich during the past 7 days. Daily intake of retinol and carotene were estimated using the Standard Tables of Food Composition in Japan (4th revised edition published by the Science and Technology Agency of Japan). The detailed information about the dietary questionnaire including validity was described elsewhere (Shimizu et al, 1996). A total of 167 cases and matched controls responded to the questionnaire.

Blood sampling as well as collection of cervical smear samples were performed on the same day. Serum was isolated and frozen at –80°C. The fraction of carotenoids, retinol and tocopherol of the serum samples were extracted into n-hexane, and the concentrations of $\alpha$-carotene, $\beta$-carotene, cryptoxanthin, lutein/zeaxanthin, lycopene, and $\alpha$-tocopherol were determined by high-performance liquid chromatography (HPLC) using a slight modification of Nells and Leenheer's method (Nells and De Leenheer, 1983). The serum of cases and matched controls was assayed for micronutrients on the same day, with the same reagent, and by the same technician. Type V $\alpha$-carotene, type IV $\beta$-carotene, lycopene purchased from Sigma Co. Ltd (St Louis, MO, USA), $\beta$-cryptoxanthin and zeaxanthin from Roche Co. Ltd (Basel, Switzerland), and $\alpha$-tocopherol from Eizai Co. Ltd (Tokyo, Japan) were used as standards. Analytical conditions for the HPLC method were described elsewhere (Ito et al, 1990).

For HPV detection, the swabs were placed into 2-ml phosphate-buffered saline (PBS). The cell suspensions after vigorous vortexing were frozen at –80°C and stored until the assay for detection of HPV. The cellular DNA was extracted by standard sodium dodecyl sulphate (SDS)-proteinase K producer. The PCR for the L1 region was done by the partly modified method previously described by Yoshikawa et al (1991). We used the consensus L1 primers L1C1 (5′-CGTAAACGTTTTCCCTATTTTTTT-3′) (1 $\mu$M), L1C2 (5′-TACCCTAAATACTCTGTATTG-3′) (0.5 $\mu$M) and L1C2M (5′-TACCCTAAATACCCTATATTG-3′) (0.5 $\mu$M) for the PCR. HPV types were identified on the basis of the restriction fragment length polymorphisms (RFLP). The PCR-based assay (L1-PCR) can type at least 26 registered genital HPVs, namely types 6, 11, 16, 18, 30, 31, 33, 34, 35, 39, 42, 43, 44, 45, 51, 52, 53, 54, 55, 56, 58, 59, 61, 66, 68 and 70.

The HPV findings and risk of cervical dysplasia based on 167 pairs have been described elsewhere (Yoshikawa et al, 1999). As we could not obtain blood samples from 12 cases and eight controls, the present analysis was restricted to 152 matched pairs with carotenoid data. These excluded cases and controls were younger than the rest of each group respectively (the mean (s.d.) ages were 32.7 (5.8) vs 40.9 (7.8) in cases and 35.8 (10.0) vs 41.0 (7.8) in controls). However, the distributions of other study variables in the excluded cases and controls were similar to those in the rest of each group after controlling for age. The distribution of diagnosis in 152 cases were 88 mild, 34 moderate and 30 severe dysplasia.

To evaluate the associations of dietary and serum micronutrients, odds ratios (ORs) and 95% confidence intervals (CIs) were estimated using conditional logistic regression model. Each micronutrient level was categorized by tertile according to the distribution in controls. Test for linear trend was obtained by assigning the median values to each category level. To distinguish the independent effect of dietary or serum micronutrient on the risk of cervical dysplasia, we calculated ORs with and without adjustment for HPV test result (negative or positive) and smoking status (current or non-smokers). Confounding effects of other variables such as number of births, age at first pregnancy, lifetime number of sexual partners and age at first intercourse were also evaluated by including them into the models. All statistical analyses were done using the SAS programs (SAS Institute, Cary, NC, USA).

### RESULTS

There were no significant differences in retinol and carotene intake between cases and controls (Table 1). Significantly lower serum levels of $\alpha$-carotene, $\beta$-carotene and lycopene were observed in cases.

In univariate analyses, there were significant inverse associations between serum levels of retinol, $\alpha$-carotene, $\beta$-carotene and lycopene and risk of cervical dysplasia (Table 2). The dose–response relationships were also statistically significant for these variables. Serum $\alpha$-carotene was significantly inversely associated with risk of cervical dysplasia after controlling for HPV infection and smoking status. The OR for the highest tertile of lycopene was lowered to 0.28 after adjustment, but was of borderline significance ($P$ = 0.06). The dose–response for decreasing risk

**Table 1** Selected characterisics of 152 matched pairs

| Variables | Cases | Controls |
|---|---|---|
| Mean (s.d.) age (years) | 40.8 (7.8) | 41.2 (7.8) |
| Mean (s.d.) number of births | 1.77 (1.13) | 2.23 (1.08)[a] |
| Marital status (%) | | |
| Married | 126 (85.7) | 112 (75.7) |
| Separated/widowed | 12 (8.2) | 16 (10.8) |
| Never-married | 9 (6.1) | 20 (13.5) |
| Cigarette smoking (%) | | |
| Never | 99 (67.4) | 109 (74.7) |
| Former | 13 (8.8) | 17 (11.6) |
| Current | 35 (23.8) | 20 (13.7) |
| Age at first intercourse (%) | | |
| –18 | 14 (9.5) | 15 (10.1) |
| 19–23 | 104 (70.3) | 86 (57.7) |
| 24+ | 30 (20.3) | 48 (32.4) |
| Lifetime number of sexual partners (%) | | |
| 0–1 | 68 (46.0) | 88 (59.1) |
| 2–3 | 47 (31.8) | 39 (26.2) |
| 4+ | 33 (22.3) | 22 (14.8) |
| HPV infection (%) | | |
| Negative | 35 (24.0) | 103 (85.1) |
| Positive | 111 (76.0) | 18 (14.9)[b] |
| Mean (s.d.) intake ($\mu$g) | | |
| Rentinol | 576 (931) | 388 (738) |
| Carotene | 4402 (3694) | 4567 (3836) |
| Mean (s.d.) serum micronutrient ($\mu$mol $l^{-1}$) | | |
| Retinol | 2.04 (0.68) | 2.14 (0.63) |
| $\alpha$-carotene | 0.15 (0.11) | 0.19 (0.13)[a] |
| $\beta$-carotene | 1.08 (0.71) | 1.34 (0.84)[a] |
| Zeaxanthin/lutein | 0.93 (0.40) | 1.02 (0.52) |
| Cryptoxanthin | 0.84 (0.95) | 0.71 (0.64) |
| Lycopene | 0.55 (0.33) | 0.65 (0.37)[a] |
| $\alpha$-tocopherol | 17.1 (5.62) | 17.3 (4.31) |

[a]$P$ < 0.01 for $t$-test. The values for serum micronutrients was log-transformed for $t$-test. [b]$P$ < 0.001 for $\chi^2$ test.

© 1999 Cancer Research Campaign

**Table 2** Odds ratios (ORs) of cervical dysplasia according to tertiles of dietary and serum micronutrients

| Variable (median) | Number of cases/controls | OR (95% CI) | Adjusted[a] OR (95% CI) |
|---|---|---|---|
| Dietary retinol ($\mu$g) | | | |
| 1 (81.4) | 50/49 | 1.00 | 1.00 |
| 2 (165.7) | 40/47 | 0.72 (0.38–1.36) | 0.51 (0.13–2.00) |
| 3 (309.4) | 56/49 | 1.05 (0.59–1.86) | 2.02 (0.62–6.60) |
| *P* for trend | | 0.70 | 0.16 |
| Dietary carotene ($\mu$g) | | | |
| 1 (1676) | 44/49 | 1.00 | 1.00 |
| 2 (3528) | 54/47 | 1.45 (0.75–2.78) | 1.47 (0.41–5.33) |
| 3 (6725) | 48/49 | 1.19 (0.61–2.31) | 2.34 (0.60–9.05) |
| *P* for trend | | 0.88 | 0.21 |
| Serum micronutrients ($\mu$mol $l^{-1}$) | | | |
| Retinol | | | |
| 1 (1.56) | 75/50 | 1.00 | 1.00 |
| 2 (2.10) | 29/50 | 0.31 (0.16–0.61) | 0.21 (0.05–0.83) |
| 3 (2.65) | 48/52 | 0.48 (0.25–0.91) | 0.50 (0.14–1.80) |
| *P* for trend | | 0.03 | 0.32 |
| $\alpha$-carotene | | | |
| 1 (0.095) | 84/50 | 1.00 | 1.00 |
| 2 (0.158) | 45/51 | 0.49 (0.27–0.89) | 0.19 (0.04–0.82) |
| 3 (0.256) | 23/51 | 0.27 (0.14–0.52) | 0.16 (0.04–0.62) |
| *P* for trend | | 0.0001 | 0.01 |
| $\beta$-carotene | | | |
| 1 (0.639) | 65/50 | 1.00 | 1.00 |
| 2 (1.063) | 57/50 | 0.89 (0.52–1.54) | 1.13 (0.42–3.03) |
| 3 (2.061) | 30/52 | 0.45 (0.25–0.82) | 0.65 (0.22–1.92) |
| *P* for trend | | 0.007 | 0.38 |
| Zeaxanthin/lutein | | | |
| 1 (0.647) | 68/51 | 1.00 | 1.00 |
| 2 (0.937) | 40/49 | 0.63 (0.36–1.08) | 0.69 (0.22–2.14) |
| 3 (1.338) | 44/52 | 0.64 (0.37–1.11) | 0.53 (0.16–1.78) |
| *P* for trend | | 0.11 | 0.31 |
| Cryptoxanthin | | | |
| 1 (0.228) | 51/51 | 1.00 | 1.00 |
| 2 (0.467) | 47/51 | 0.92 (0.52–1.63) | 0.86 (0.29–2.57) |
| 3 (1.256) | 54/50 | 1.10 (0.61–1.97) | 1.35 (0.46–4.02) |
| *P* for trend | | 0.66 | 0.56 |
| Lycopene | | | |
| 1 (0.307) | 69/51 | 1.00 | 1.00 |
| 2 (0.573) | 45/51 | 0.58 (0.31–1.06) | 0.53 (0.15–1.90) |
| 3 (0.973) | 38/50 | 0.53 (0.30–0.95) | 0.28 (0.08–1.01) |
| *P* for trend | | 0.047 | 0.049 |
| $\alpha$-tocopherol | | | |
| 1 (13.8) | 56/51 | 1.00 | 1.00 |
| 2 (16.9) | 52/51 | 0.91 (0.53–1.59) | 0.56 (0.18–1.96) |
| 3 (20.8) | 44/50 | 0.78 (0.44–1.41) | 0.85 (0.28–2.61) |
| *P* for trend | | 0.68 | 0.80 |

[a]Adjusted for HPV infection and smoking.

with increasing lycopene level was slightly statistically significant. The association of $\beta$-carotene with risk of cervical dysplasia was not statistically significant after controlling for HPV infection and smoking status.

Additional adjustments for number of births, marital status and variables for sexual behaviours did not alter the results substantially.

We further categorized HPV infection status according to HPV type; high-risk HPVs (types 16 and 18), intermediate-risk HPVs (types 31, 33, 51, 52, 56, 58, 59, 68 and 70) and low-risk HPVs (types 6, 11, 42, 53, 54, 61 and 66 or unknown types). Adjusting for HPV type instead of HPV positivity did not change the results substantially (for example, the ORs for the second and the highest tertiles of serum $\alpha$-carotene were 0.23 and 0.17, respectively, after controlling for HPV type and smoking status).

### DISCUSSION

We found that high levels of serum $\alpha$-carotene and lycopene were associated with a decreased risk of cervical dysplasia. These associations were not explained by confounding due to HPV infection and other factors. We had to exclude 11 cases and 35 controls without data on HPV infection or smoking status from the multivariate analyses. However, the results of univariate analyses excluding them were similar to those based on the entire subjects.

The present study supports the hypothesis that carotenoids play an important role in the genesis of cervical cancer. To our knowledge, only three studies assessed the association between serum micronutrients and risk of cervical neoplasia after allowing for HPV infection. Potishman et al (1991) measured the same serum markers as we did, but found that only $\beta$-carotene was significantly

© 1999 Cancer Research Campaign inversely associated with risk of invasive cervical cancer. In their study, HPV infection was detected by filter in situ hybridization, as used in early studies, which is less sensitive than the PCR method (Schiffman, 1992). In the other two studies, Butterworth et al (1992) measured only total carotenoids and Kwañniewska et al (1996) measured only $\beta$-carotene. Reduced risk associated with higher serum $\alpha$-carotene was observed in the studies by VanEenwyk et al (1991) and Batieha et al (1993), although HPV infection was not adjusted for. Serum lycopene was significantly inversely associated with risk of cervical neoplasia in these studies.

The multivariate analyses in our study showed that confounding effect of HPV infection was not so great except that for the association of $\beta$-carotene with risk of cervical dysplasia. Adjusting for HPV infection and smoking status somewhat strengthened the associations for retinol, $\alpha$-carotene, and lycopene. Most previous studies which found a negative association between serum micronutrients and cervical neoplasia did not consider the effect of HPV infection (Bernstein and Harris, 1984; Harris et al, 1986; Brock et al, 1988; Palan et al, 1988; Verreault et al, 1989; VanEenwyk et al, 1991; Batieha et al, 1993).

$\beta$-carotene as well as other carotenoids act as antioxidants (Burton and Ingold, 1984), suggesting their potential usefulness in disease prevention. Although data from cervical cancer chemoprevention trials failed to show a preventive effect of $\beta$-carotene (Romney et al, 1997), other carotenoids such as $\alpha$-carotene and lycopene may prevent cervical cancer. It is also possible that $\beta$-carotene may not work after the genesis of cervical dysplasia.

As the sera were not obtained from cases before the diagnosis of cervical dysplasia, we cannot exclude the possibility that the observed associations may be a consequence of the disease rather than a reflection of its aetiology.

High intake of retinol and carotene were associated with increased risk of cervical dysplasia, although they did not attain statistical significance. If these are true, decreased serum carotenoid in cases could not be explained by low intake of foods rich in carotenoids. These findings also suggest that decrease in serum carotenoids is a result rather a cause of disease. On the other hand, there is a possibility that the observed positive associations for dietary retinol and carotene are due to recall bias. It is also possible that the assessment of nutrient intake was based on intake amounts during the past week which may not have accurately reflected dietary intakes at the relevant time.

### ACKNOWLEDGEMENTS

This work was supported by cancer research grants from the Ministry of Education, Science, Sports, and Culture, Japan (A03-08264103, X0010150101).

### REFERENCES

Batieha AW, Armenian HK, Norkus EP, Morris JS, Spate VE and Comstock GW (1993) Serum micronutrients and the subsequent risk of cervical cancer in a population-based nested case–control study. *Cancer Epidemiol Biomarkers Prev* **2**: 335–339

Bernstein A and Harris B (1984) The relationship of dietary and serum vitamin A to the occurrence of cervical intraepithelial neoplasia in sexually active women. *Am J Obstet Gynecol* **148**: 309–312

Brock KE, Mock GBP, Maclennan R, Truswell AS and Brinton LA (1988) Nutrients in diet and plasma and risk of in situ cervical cancer. *J Natl Cancer Inst* **80**: 580–585

Burton GW and Ingold KU (1984) $\beta$-carotene: an unusual type of lipid antioxidant. *Science* **224**: 569–573

Butterworth CE, Hatch KD, Macaluso M, Cole P, Sauberlich HE, Soong S, Borst M and Baker VV (1992) Folate deficiency and cervical dysplasia. *J Am Med Assoc* **267**: 528–533

Cuzick J, De Stavola BL, Russell MJ and Thomas BS (1990) Vitamin A, vitamin E and the risk of cervical intraepithelial neoplasia. *Br J Cancer* **62**: 651–652

De Vet HCW, Knipschild PG, Grol MEC, Schouten HJA and Sturmans AF (1991) The role of beta-carotene and other dietary factors in the aetiology of cervical dysplasia: results of a case–control study. *Int J Epidemiol* **20**: 603–610

Harris RWC, Forman D, Doll R, Vessey MP and Wald NJ (1986) Cancer of the cervix uteri and vitamin A. *Br J Cancer* **53**, 653–659

Herrero R (1996) Epidemiology of cervical cancer. *Monogr Natl Cancer Inst* **21**: 1–6

Herrero R, Potishman N, Brinton LA, Reeves WC, Brenes MM, Tenorio F, De Brintton RC and Gaitan E (1991) A case–control study of nutrient status and invasive cervical cancer. *Am J Epidemiol* **134**: 1335–1346

Ito Y, Ochiai J, Sasaki R, Suzuki S, Kusuhara Y, Morimitsu Y, Otani M and Aoki K (1990) Serum concentrations of carotenoids, retinol, and $\alpha$-tocopherol in healthy persons determined by high-performance liquid chromatograohy. *Clin Chim Acta* **194**: 131–144

Kwañniewska A, Yukendorf A and Semczuk M (1996) Content of $\beta$-carotene in blood serum of human papillomavirus infected women with cervical dysplasia. *Arch Immunol Ther Exp* **44**: 309–313

La Vecchia C, Decarli A, Fasoli M, Parazzini F, Franceschi S, Gentlie A and Negri E (1988) Dietary vitamin A and risk of intraepithelial and invasive cervical neoplasia. *Gynecol Oncol* **30**: 187–195

Marshall JR, Graham S, Byers T, Swanson M and Brasure J (1983) Diet and smoking in the epidemiology of cancer of the cervix. *J Natl Cancer Inst* **70**: 847–851

Nells HJCF and De Leenheer AP (1983) Isocratic noaquenous reversed-phase liquid chromatography of carotenoids. *Anal Chem* **55**: 270–275

Palan PR, Romney SL, Nikham M, Basu J and Vermund SH (1988) Decreased plasma beta-carotene levels in women with uterine cervical dysplasias and cancer. *J Natl Cancer Inst* **80**: 454–455

Palan PR, Mikhail MS, Basu J and Romney SL (1991) Plasma levels of antioxidant $\beta$-carotene and $\alpha$-tocopherol in uterine cervix dysplasia and cancer. *Nutr Cancer* **15**: 13–20

Potischman N and Brinton LA (1996) Nutrition and cervical neoplasia. *Cancer Causes Control* **7**: 113–126

Potishman N, Herrero R, Brinton LA, Reeves WC, Staceicz-Spauntzakis M, Jones CJ, Brenes MM, Tenorio F, de Britton RC and Gaitan E (1991) A case–control study of nutrient status and invasive cervical cancer. II. Serological indicators. *Am J Epidemiol* **134**: 1347–1355

Romney SL, Ho GYF, Palan PR, Basu J, Kadish AS, Klein S, Mikhail M, Hagan RJ, Chang CJ and Burk RD (1997) Effects of $\beta$-carotene and other factors on outcome of cervical dysplasia and human papillomavirus infection. *Gynecol Oncol* **65**: 483–492

Schiffman MH (1992) Validation of hybridization assays: correlation of filter in situ, dot blot and PCR with Southern blot. In: *The Epidemiology of Human Papillomavirus and Cervical Cancer*, Mujoz N, Bosch FX, Shah KV and Meheus A (eds), pp. 169–179. IARC Scientific Publication No. 119: Lyon

Schneider A and Shah K (1989) The role of vitamins in the etiology of cervical neoplasia: an epidemiological review. *Arch Gynecol Obstet* **246**: 1–13

Shimizu H, Nagata C, Komatsu S, Morita N, Higashiiwai H, Sugahara N and Hisamichi S (1996) Decreased serum retinol levels in women with cervical dysplasia. *Br J Cancer* **73**: 1600–1604

VanEenwyk J, Davis FG and Bowen P (1991) Dietary and serum carotenoids and cervical intraepithelial neoplasia. *Int J Cancer* **48**: 34–38

Verreault R, Chu J, Mandelson M and Shy K (1989) A case–control study and invasive cervical cancer. *Int J Cancer* **43**: 1050–1054

Yoshikawa H, Kawana T, Kitagawa K, Mizuno M, Yoshikura H and Iwamoto A (1991) Detection and typing of multiple genital human papillomaviruses by DNA amplification with consensus primers. *Jpn J Cancer Res* **82**: 524–531

Yoshikawa H, Nagata C, Noda K, Nozawa S, Yajima A, Sekiya S, Sugimori H, Hirai Y, Kanazawa K, Sugase M, Shimizu H and Kawana T (1999) Human papillomavirus-independent risk factors for cervical intraepithelial neoplasia in Japan. *Br J Cancer* (in press)

Ziegler RG, Brinton LA, Hamman RF, Lehman HF, Levine RS, Mallin LK, Norman SA, Rosenthal JF, Trumble AC and Hoover RN (1990) Diet and the risk of invasive cervical cancer among white women in the United States. *Am J Epidemiol* **132**: 432–445

*© 1999 Cancer Research Campaign*
